# Preparation and Characterization of Highly Fluorescent, Glutathione-coated Near Infrared Quantum Dots for *in Vivo* Fluorescence Imaging

**DOI:** 10.3390/ijms9102044

**Published:** 2008-10-29

**Authors:** Takashi Jin, Fumihiko Fujii, Yutaka Komai, Junji Seki, Akitoshi Seiyama, Yoshichika Yoshioka

**Affiliations:** 1 WPI Immunology Frontier Research Center, Osaka University, Suita 565–0871, Osaka, Japan. E-Mails: ffujii@fbs.osaka-u.ac.jp (F. F.); ykomai@fbs.osaka-u.ac.jp (Y. K.); sekij@ri.ncvc.go.jp (J. S.); yoshioka@fbs.osaka-u.ac.jp (Y. Y.); 2 Graduate School of Frontier Biosciences, High Performance Bioimaging Facility, Osaka University, Suita 565–0871, Osaka, Japan; 3 National Cardiovascular Center Research Institute, Suita 565–8561, Osaka, Japan; 4 Graduate School of Medicine, Kyoto University, Kyoto 606–8507, Japan. E-Mail: aseiyama@hs.med.kyoto-u.ac.jp (A. S.)

**Keywords:** Near-infrared quantum dots, glutathione, ligand-exchange, cytotoxicity, *in vivo* fluorescence imaging

## Abstract

Fluorescent probes that emit in the near-infrared (NIR, 700–1,300 nm) region are suitable as optical contrast agents for *in vivo* fluorescence imaging because of low scattering and absorption of the NIR light in tissues. Recently, NIR quantum dots (QDs) have become a new class of fluorescent materials that can be used for *in vivo* imaging. Compared with traditional organic fluorescent dyes, QDs have several unique advantages such as size- and composition-tunable emission, high brightness, narrow emission bands, large Stokes shifts, and high resistance to photobleaching. In this paper, we report a facile method for the preparation of highly fluorescent, water-soluble glutathione (GSH)-coated NIR QDs for *in vivo* imaging. GSH-coated NIR QDs (GSH-QDs) were prepared by surface modification of hydrophobic CdSeTe/CdS (core/shell) QDs. The hydrophobic surface of the CdSeTe/CdS QDs was exchanged with GSH in tetrahydrofuran-water. The resulting GSH-QDs were monodisperse particles and stable in PBS (phosphate buffered saline, pH = 7.4). The GSH-QDs (800 nm emission) were highly fluorescent in aqueous solutions (quantum yield = 22% in PBS buffer), and their hydrodynamic diameter was less than 10 nm, which is comparable to the size of proteins. The cellular uptake and viability for the GSH-QDs were examined using HeLa and HEK 293 cells. When the cells were incubated with aqueous solutions of the GSH-QDs (10 nM), the QDs were taken into the cells and distributed in the perinuclear region of both cells. After 12 hrs incubation of 4 nM of GSH-QDs, the viabilities of HeLa and HEK 293 cells were ca. 80 and 50%, respectively. As a biomedical utility of the GSH-QDs, *in vivo* NIR-fluorescence imaging of a lymph node in a mouse is presented.

## 1. Introduction

In living tissues, intrinsic chromophores like hemoglobin and water are the major absorbers of visible and infrared light [1]. Near infrared (NIR) light ranging from 700 to 1,300 nm can penetrate into deeper tissues (ca. 0.5 mm to cm) because of the low absorbance and scattering in the tissues [1–3]. The NIR region is called as “the optical window” for *in vivo* imaging at a whole body level [1]. For *in vivo* fluorescence imaging, NIR fluorophores can be used as optical contrast agents. So far, traditional fluorescent dyes such as Cy7, oxazine 750, and indocyanine green (ICG) have been used as NIR-fluorescent probes for *in vivo* imaging [4–5]. However, traditional NIR-dyes have several disadvantages for use as fluorescent probes: low solubility in aqueous solution, low quantum yield, and low photostability. For example, ICG, the most widely used NIR-dye only provides a quantum yield of 1.2% [6] in blood. In addition, the photostability of ICG is very poor and the fluorescence of ICG in aqueous solution diminishes within several days under room right [7].

Recent developments in synthetic techniques for NIR-emitting QDs have paved the way for use of NIR QDs as fluorescence contrast agents for *in vivo* imaging [8–16]. In comparison with organic NIR dyes, NIR QDs are highly bright and resistant to photobleaching [11, 13]. Kim *et al.* first reported the biological utility of NIR QDs (CdTe/CdSe) for sentinel lymph node imaging in a mouse and pig [17]. Morgan *et al.* demonstrated that the NIR QDs (CdMnTe/Hg) are useful as angiographic contrast agents for vessels surrounding and penetrating a cell carcinoma in a mouse [18]. So far, a number of highly fluorescent NIR QDs such as PbS [19–21], CdSeTe/CdS [22–24], CdHgTe/ZnS [25], and InAs/CdSe/ZnSe [26] have been reported. These QDs are mostly synthesized by heating appropriate organometallic precursors in organic solvents [e.g. trioctylphosphine oxide (TOPO) and trioctylphosphine (TOP)] [27–28]. Thus the resulting NIR QDs are very hydrophobic and insoluble to water. For the application of NIR QDs for *in vivo* imaging, the hydrophobic surface of the QDs should be modified to be hydrophilic. The surface coating of QDs strongly affects the fluorescence intensity, particle size, and stability of QDs in aqueous solution [29–31]. The surface coating also affects the cytotoxicity of QDs. It has been shown that mercaptopropionic acid (MPA)-coated CdSe/ZnS QDs show cytotoxicity to adherent cells at high particle concentrations, while silane-coated QDs do not affect the viability of the cells [32].

The objective of this work was to develop a facile method for the preparation of highly fluorescent NIR QDs that can be used for *in vivo* fluorescence imaging. Although there have been a number of reports regarding the synthesis of hydrophobic NIR QDs, the preparation of biocompatible NIR QDs that emit at 700–1,300 nm is still challenging, as surface modification of QDs often results in a dramatic loss of both fluorescence and a long-term stability of the QDs because of the degradation of the QD surface or desorption of stabilizing organic layers surrounding QDs. In this paper, we report the preparation and characterization of glutathione (GSH)-coated biocompatible NIR QDs with a high quantum yield (22%) and a long-term stability (> 1 month) in neutral aqueous solution. The cell viability for the GSH-coated NIR QDs and *in vivo* lymph node imaging using the QDs in a mouse are also reported.

## 2. Results and Discussion

### 2.1. Fluorescence and absorption spectra of hydrophobic CdSeTe/CdS QDs

**Figure 1.**
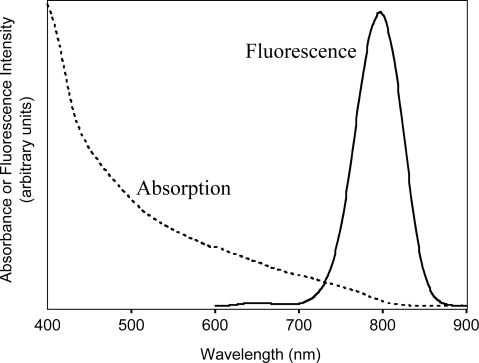
Fluorescence and absorption spectra of CdSeTe/CdS QDs in chloroform. Fluorescence spectrum was measured at excitation of 480 nm.

First, as highly fluorescent hydrophobic NIR QDs, CdSeTe/CdS QDs with a core-shell structure were synthesized by a modified method based on the procedure reported by Bailey *et al.* [22–23]. Figure 1 shows the fluorescence and absorption spectra of CdSeTe/CdS QDs in chloroform.

The fluorescence spectrum of the QDs (800 nm emission) shows a symmetrical shape with a spectral width (the full width at half maximum, fwhm) of 65 nm. In the synthesis of the CdSeTe/CdS QDs, we could control the emission maximum ranging from 700 to 850 nm by changing the reaction time for the growth of the QDs or the injection temperature of a Se-Te precursor [22–24]. Compared with other types of NIR QDs such as CdTe/CdSe and CdHgTe/CdS, the fwhm of the CdSeTe/CdS QDs is narrow: in the case of CdTe/CdSe QDs (800 nm emission), the fwhm is ca. 100 nm [33]. The fwhm of CdHgTe/CdS QDs (771 nm emission) is reported as 76 nm [34]. In addition, the quantum yield (QY) of CdSeTe/CdS QDs is very high and the value is determined to be 36% in chloroform as a reference standard of ICG in dimethylsulfoxide, DMSO (QY = 12%) [6]. The absorption spectrum of the CdSeTe/CdS QDs is very broad and the QDs absorb the light from visible to NIR region (700 to 800 nm). Thus the CdSeTe/CdS QDs can be excited by the NIR light as well as visible light.

### 2.2. Surface coating of hydrophobic CdSeTe/CdS QDs with glutathione (GSH)

So far, GSH-coated QDs have been prepared by aqueous synthetic methods, where GSH is used as a stabilizing agent for QDs in aqueous solution [35–37]. Recently, Gill *et al.* reported water-soluble GSH-coated QDs prepared by a ligand exchange of hydrophobic CdSe/ZnS QDs [38]. In this work, we modified the surface of hydrophobic CdSeTe/CdS QDs with GSH to solubilize them in water.

Water-solubilization of CdSeTe/CdS QDs by surface coating with thiol compounds has been reported by two groups. Bailey *et al.* reported that CdSeTe/CdS QDs can be made water-soluble by mercaptoacetic acid (MAA) coating and the fluorescence brightness of the QDs retains after water-solubilization [23]. Jiang, *et al.* also reported mercaptoundecanoic acid (MUA) coating for the preparation of highly fluorescent water-soluble CdSeTe/CdS QDs [24]. Although these coating methods are simple and convenient for the preparation of water-soluble CdSeTe/CdS QDs, the resulting QDs are not stable in neutral aqueous solutions. In the case of MAA-coated CdSeTe/CdS QDs, the QDs precipitate out of solution after 2–3 weeks [23]. To overcome this problem, we developed a new coating method using a natural thiol compound, glutathione (GSH). GSH is a tripeptide (©-l-glutamyl-l-cysteinylglycine) that exists in most organs, and the compound is not harmful like MAA and MUA. In addition, GSH is known to have a property to detoxify Cd^2+^ ions in cellular level due to its chelating capability [39].

**Figure 2.**
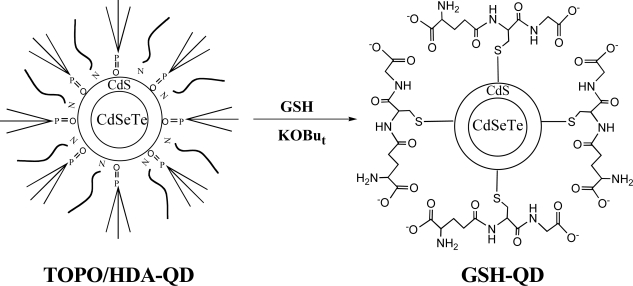
Schematic representation for the preparation of GSH-coated QDs. GSH coating is performed in a mixture of THF-water at 60 ºC. Potassium *t*-_butoxide (KOBu_*_t_*) _is used as_ a deprotonation reagent.

Figure 2 shows schematic representation for the surface coating of CdSeTe/CdS QDs with GSH. First, the hydrophobic CdSeTe/CdS QDs surrounding trioctylphosphine oxide (TOPO) and hexadecylamine (HDA) molecules are dispersed to tetrahydrofuran (THF). Then an aqueous solution of GSH is added to the THF solution of QDs and the ligand-exchange is performed at 60 ºC. After the deprotonation of the carboxyl groups of GSH with potassium *t*-butoxide (KOBu*t*), GSH-coated CdSeTe/CdS QDs are dissolved easily in water.

Since the GSH-coated CdSeTe/CdS QDs have carboxyl groups at the surface, the conjugation of proteins and antibodies is easily carried out in the presence of cross-coupling agents such as EDC (1-ethyl-3-(3-dimethylaminopropyl)-carbodiimide) [40]. In addition, since the GSH-coated QDs have amino groups at the surface, a variety of amine reactive reagents such as NHS esters, isothiocyanates and isocyanates [36] can be used for the surface modification of the GSH-QDs.

### 2.3. Fluorescence brightness of GSH-coated CdSeTe/CdS QD and its stability in PBS buffer

**Figure 3.**
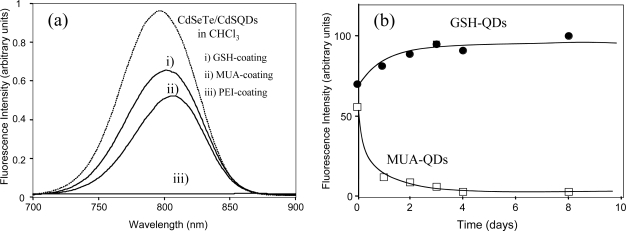
(a) Fluorescence spectra of CdSeTe/CdS QDs before and after surface modification with GSH, MUA and PEI. The spectra of GSH-, MUA- and PEI-coated QDs were measured in 10 mM PBS buffer. Excitation was performed at 480 nm. The absorbance at 480 nm was set to be 0.05 OD for all samples. (b) Changes in the fluorescence intensity (800 nm) of the GSH- and MUA-coated CdSeTe/CdS QDs (GSH-QDs an MUA-QDs) in 10 mM PBS buffer (pH = 7.4).

Figure 3a shows the fluorescence spectra of CdSeTe/CdS QDs before and after surface modification with GSH, MUA and polyethyleneimine (PEI). After the surface coating, the fluorescence intensity at 800 nm in PBS buffer decreased to be 62% and 51% for GSH- and MUA-coated QDs, respectively. As a reference standard of ICG in DMSO, the quantum yield of as-prepared GSH- and MUA-coated QDs in PBS buffer were determined to be 22% and 19%, respectively. In the case of PEI-coating, the fluorescence intensity completely diminished, suggesting that degradation of the surface of QDs occurred. The stability of GSH- and MUA-coated QDs in PBS buffer was examined by the time-course of the fluorescence intensities of the QDs (Figure 3b). In the case of the GSH-coated QDs (GSH-QDs), the fluorescence intensity gradually increased and became constant after 2–3 days. The GSH-QDs were stable in PBS buffer over one month in the dark. In contrast, the fluorescence intensity of MUA-coated QDs (MUA-QDs) significantly decreased within one day. QDs coated with mono-thiol compounds such as MAA and MUA are known to be unstable in neutral solutions due to the aggregation of the QDs or desorption of the thiol compounds from the QD surface by the oxidative formation of disulfide compounds [41].

It is known that high concentrations of reduced glutathione (1–5 mM) exist in living tissues [42, 43]. For example, the concentration of GSH in the human brain is ca. 2 mM [43]. Thus, for the application GSH-QDs for *in vivo* imaging, it is important to check the stability of the QDs in physiological buffer solution in the presence of mM level of GSH.

**Figure 4.**
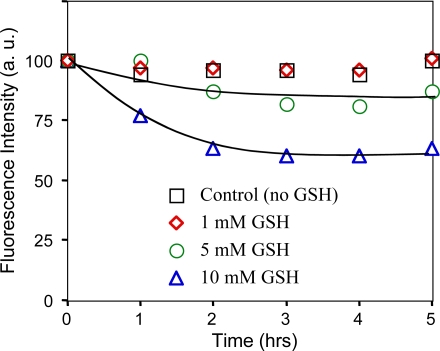
Changes in the fluorescence intensity of GSH-QDs (20 nM in 100 mM PBS buffer) in the presence of 0, 1, 5, and 10 mM GSH.

Figure 4 shows changes in the fluorescence intensity of GSH-QDs in 100 mM PBS buffer in the presence of 0, 1, 5, and 10 mM GSH. In the presence of 1mM of GSH, fluorescence intensity of the GSH-QDs was almost the same as that of the control, where no GSH was contained in the solution. The higher concentration of GSH (5 and 10 mM) caused significant decrease (20–40%) in the fluorescence intensity of the GSH-QDs. This decrease suggests degradation of the surface of the GSH-coated QDs, that may have occurred during the ligand exchange of GSH at the QD surface. However, after 3hrs, fluorescence intensity of the GSH-QDs became constant, suggesting the possible use of the QDs for cellular and *in vivo* imaging.

### 2.4. Hydrodynamic diameter and dispersibility of GSH-coated CdSeTe/CdS QDs in PBS buffer

Hydrodynamic diameter and dispersibility of QDs are important properties for their application to *in vivo* fluorescence imaging. In live animals, particle size can affect biodistribution and pharmacokinetics [44]. For example, the particle size of QDs affects endocytosis or limits access to receptors of interest on cellar membranes [45–47].

**Figure 5.**
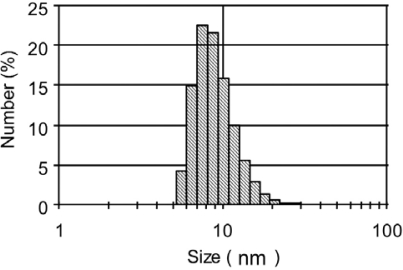
Dynamic light scattering histogram for GSH-QDs in 10 mM PBS buffer (pH = 7.4).

Figure 5 shows a dynamic light scattering (DLS) histogram for GSH-QDs in PBS buffer. The hydrodynamic size is found to be 7.2 ± 2.4 nm and this size is much smaller than that of QDs with amphiphilic polymer coatings (e. g. commercial available NIR QDs with 800 nm emission, 16.9 ± 7.3 nm in diameter [48]).

The hydrodynamic diameter of the GSH-QDs was also determined by using fluorescence correlation spectroscopy (FCS) [31,49–50]. FCS uses the fluctuations of the fluorescence intensity in a tiny confocal volume to determine the diffusion times of fluorescent particles. Fluctuations in the fluorescence intensities *I(t)* can be analyzed using the autocorrelation function *G(τ)*:

(1) $$G(\tau )=\frac{<I(t)I(\tau +t)>}{<I(t){>}^{2}}$$

where the symbol < > stands for the ensemble average.

**Figure 6.**
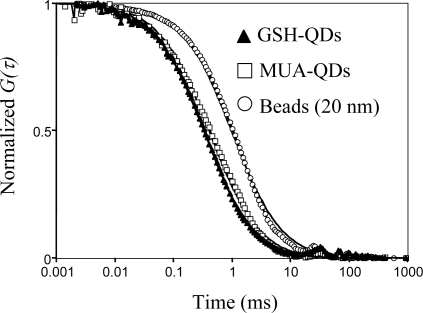
Normalized fluorescence autocorrelation curves for GSH-QDs, MUA-QDs, and fluorescent beads (20 nm in diameter) in 10 mM PBS buffer. The autocorrelation curves (solid lines) are fitted by using a one-component diffusion model, eq. (2).

If a three-dimensional Gaussian profile in the confocal volume of the lateral radius ω_0_ and axial radius ω_z_ was assumed, eq. (1) for a one-component diffusion can be expressed as:

(2) $$G(\tau )=1+\frac{1}{N}{\left(1+\frac{\tau }{\tau_{d}}\right)}^{-1}{\left(1+\frac{\tau }{{\left(\omega_{z}/2\omega_{o}\right)}^{2}\tau_{d}}\right)}^{-1/2}$$

where $\tau_{d}=\frac{\omega_{o}^{2}}{4D}$*N* is the average number of fluorescent particles in the excitation volume and *τ_d_* is the diffusion time of the fluorescent particles, depending on the diffusion constant *D* and ω*_o_*. Using the Stokes-Einstein relationship (*D=k_B_T/6*πη*r*), the hydrodynamic diameter *d_QD_* of QDs can be calculated from the following equation:

(3) $$d_{QD}=d_{ST}\times \frac{\tau_{QD}}{\tau_{ST}}$$

where *d_ST_* is the diameter of a standard particle, and *τ_QD_* and *τ_ST_* are the diffusion times of QDs and the standard particles, respectively. As a standard particle, we used a fluorescent latex bead (20 nm in diameter, Molecular Probe, Inc.). Figure 6 shows the normalized fluorescence autocorrelation curves *G(τ)* for GSH- and MUA-QDs including the *G(τ)* curve for fluorescent latex beads. All the *G(τ)* curves fit a one-component diffusion model [eq. (2)]. From the fitting curves, the diffusion times are determined to be 0.34, 0.42, and 1.0 ms for GSH-QDs, MUA-QDs, and fluorescent beads, respectively. Using eq. (3), the hydrodynamic diameter of the GSH-QDs is calculated to be 6.8 ± 0.5 nm. This value is in a good agreement with the result obtained from the DLS measurement described above. The results of DLS and FCS measurements show that the GSH-QDs exist in monodisperse particles in PBS buffer.

### 2.5. Cellular uptake and cell viability of GSH-coated CdSeTe/CdS QDs

**Figure 7.**
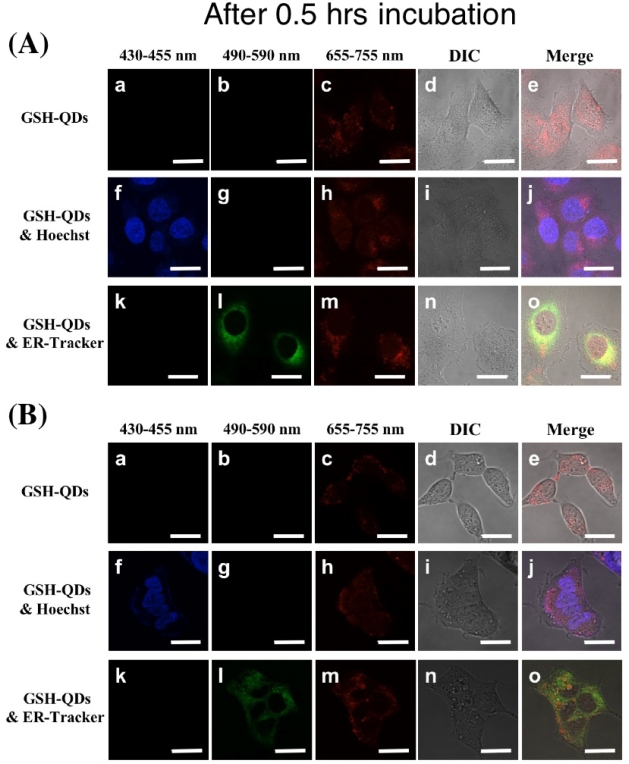

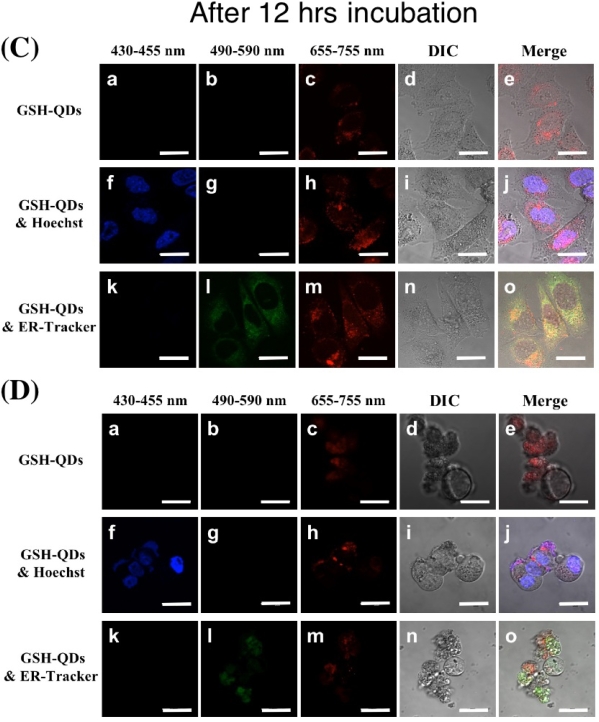
Confocal microscopy images of HeLa (A, C) and HEK 293 (B, D) cells in 0.5 and 24 hrs after treating with 10 nM GSH-QDs (top row: a-e), 10 nM GSH-QDs and 1.6 ng/mL Hoechst 33342 (middle row: f-j), and 10 nM GSH-QDs and 0.2 nM ER-Tracker^TM^ Green (bottom row: k-o). Hoechst 33342 and ER-Tracker^TM^ Green stain nuclei and endoplasmic reticulum, respectively. Scale bars in figures show 20 μm length.

We have examined the cellular uptake of GSH-QDs in cultured cells. Figure 7 shows confocal microscopy images of HeLa and HEK 293 cells treated with 10 nM of GSH-QDs for 0.5 and 12 hrs incubation. As shown in Figure 7 (A-e, B-e, C-e, D-e), the QDs enter into the both cells through endocytosis or macropinocytosis after 0.5 and 12 hrs incubation. It should be noted that the fluorescence intensity of the QDs after 12 hrs incubation (C-e, D-e) are similar to that of the QDs after 0.5 hrs incubation (A-e, B-e). This finding suggests the amounts of the QDs taken up were saturated within 0.5 hrs and the significant degradation of the QDs did occur in the cells up to 12 hrs.

**Figure 8.**
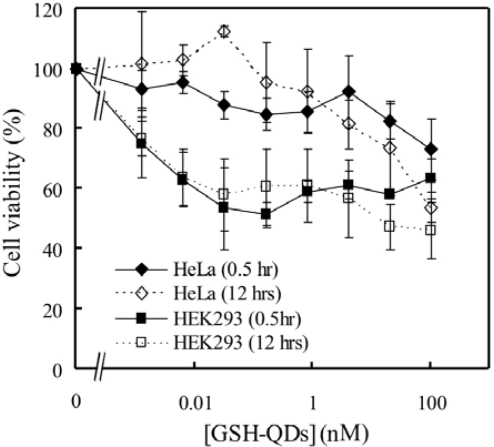
The viability of HeLa and HEK 293 cells in 0.5 and 12 hrs after treating with from 1.3 pM to 100 nM of GSH-QDs. Error bars indicate the standard deviations of three independent experiments.

To clarify the accumulated sites of the QDs in the cells, the cells were labeled simultaneously with Hoechst 33342 (A-j, B-j, C-j, D-j) or ER-Tracker^TM^ Green (A-o, B-o, C-o, D-o), which stain nuclei and endoplasmic reticulum, respectively. After 0.5 hrs incubation, the majority of the QDs were distributed in the perinuclear region (A-j, B-j) and associated with endoplasmic reticulum (A-o and Bo). The localization pattern of the QDs in HeLa cells after 12 hrs incubation (C-j) were almost the same as that after 0.5 hrs incubation (A-j). In the case of HEK 293 cells, their shapes changed to be abnormal after 12 hrs incubation (D-e), showing that the viability of HEK 293 decreased to be 50% at 10 nM of the QDs (Figure 8). The cytotoxic effects of GSH-QDs on cells are therefore different among cell types.

We have investigated the cytotoxic effects of the GSH-QDs on cultured cells. The HeLa and HEK 293 cells were incubated 0.5 and 12 hrs after treating with the GSH-QDs and then a QMTT reduction assay was performed. Figure 8 shows the dose-response effect of the GSH-QDs on the viability of HeLa and HEK 293 cells. The viability of HeLa cells after 0.5 and 12 hrs incubation remaines over 80% up to 20 nM. In contrast, the viability of HEK 293 cells decreases in dose-related manner and reached about 60% at 6.4 pM of the GSH-QDs after both 0.5 and 12 hrs incubation.

### 2.6. In vivo lymph node imaging using GSH-coated CdSeTe/CdS QDs

**Figure 9.**
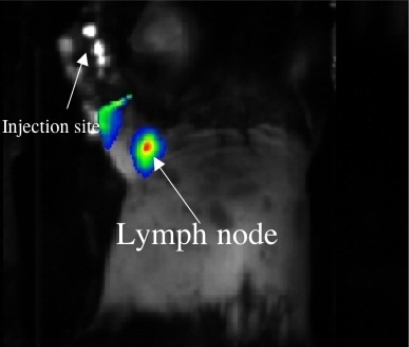
*In vivo* imaging for a lymph node in a mouse. NIR-light was illuminated by an array of the laser diodes with a 785 nm band-pass filter, and the NIR-fluorescence was detected by a cooled CCD (PIXIS-2048B, Roper Industries) with an 845 nm band-pass filter. The time of NIR-illumination was 10 sec. A pseudo-colored fluorescence image was superimposed on a monochrome visible light image.

We tested whether GSH-coated NIR QDs can be applied to *in vivo* lymph node imaging. Kim *et al.* first used NIR QDs for *in vivo* imaging of sentinel lymph node mapping in animals [17]. They injected type II QDs (CdTe/CdSe) of 850 nm emitting (QY= 13% in PBS) intradermaly into a mouse and a pig, and could image their sentinel lymph nodes. The work demonstrated that NIR-imaging of QDs could serve surgical guide in tumor excision. Figure 9 shows a pseudo-colored NIR-image of a lymph node in a mouse using the GSH-coated NIR QDs, where QDs (100 μl of 1μM) were injected from the right fore-palm with a syringe. The QDs migrated to a nearby lymph node, and the lymph node could be imaged virtually background free. This image clearly demonstrates that the injection of at least 100 pmol of the GSH-coated NIR QDs can visualize a lymph node in a mouse.

## 3. Conclusions

In this paper, we have presented a facile method for the preparation of highly fluorescent, GSH-coated NIR QDs (CdSeTe/CdS) with a core-shell structure. GSH is a natural thiol compound and not a harmful reagents such as synthetic thiol compounds, MAA and MUA. Compared with MAA- and MUA-coating, GSH-coating results in highly stable QDs in aqueous solution. In addition, the size of GSH-coated NIR QDs is very compact (< 10 nm in diameter), and the colloidal stability of the QDs in PBS buffer (pH = 7.4) is much better than that of MAA- and MUA-coated QDs. The GSH-coated NIR QDs can be used for NIR fluorescence imaging at cellular and whole body level. In the HeLa and HEK 293 cells, the GSH-coated NIR QDs are taken into the cells and distributed in their perinuclear region. For 12 hrs incubation of 4 nM of the QDs, the viabilities of HeLa and HEK293 cells were observed to be ca. 80 and 50%, respectively. The biological utility of the GSH-coated NIR QDs was demonstrated for *in vivo* lymph node imaging using pmol level of the QDs. The GSH-coated NIR QDs have both carboxyl and amino groups at the surface. Thus a number of bioconjugate techniques can be used for functionalization of the QDs. We expect that GSH-coated CdSeTe/CdS QDs can be applied as biocompatible NIR fluorescent probes for *in vivo* fluorescence imaging.

## 4. Experimental Section

### 4.1. Materials

Cadmium oxide (CdO, 99.99%), Selenium (Se, powder, 99. 999%), Tellurium (Te, shot, 1–2 mm, 99.99%), indocyanine green (ICG), mercaptoundecanoic acid (MUA), and polyethyleneimine (PEI, MW. 10,000) were purchased from Sigma-Aldrich. Tri-octylphosphine oxide (TOPO), tri-octylphosphine (TOP), tri-butylphosphine (TBP), hexadecylamine (HDA, 90%), and stearic acid were purchased from Tokyo Chemical Industry (Japan). Sulfur (S, crystalline, 99.9999%), glutathione (GSH, reduced form) and potassium *t*-butoxide were purchased from Wako (Japan). Other organic solvents used were of analytical reagent grades.

Stock solutions were prepared and stored under an argon atmosphere. A Se-Te stock solution was prepared by dissolving Se (24 mg, 0.3 mmol) and Te (13 mg, 0.1 mmol) in TBP (1 mL) at room temperature. A Cd-S stock solution was prepared as follows: sulfur (40 mg, 1.25 mmol) was added to TOP (10 mL) and heated at 100 ºC. After sulfur was completely dissolved, the solution was cooled to room temperature. A mixture of CdO (160 mg, 1.25 mmol) and stearic acid (2 g) was loaded into a 25 mL three-necked flask and heated at 300 ºC. After CdO was completely dissolved, the solution was cooled to 80 ºC, and a Sulfur-TOP solution was added to the Cd-TOP solution under stirring. The Cd-S stock solution was stored under an argon atmosphere at room temperature.

### 4.2. Synthesis of CdSeTe/CdS QDs

CdSeTe/CdS QDs were synthesized by a modified method based on the reported procedure [22–23, 51–52]. A mixture of CdO (25 mg, 0.2 mmol) and stearic acid (200 mg) was loaded into a 25 mL of three-necked flask and heated at 300 ºC. After CdO was completely dissolved, the mixture cooled to room temperature. Then TOPO (2 g) and HDA (2 g) were added to the flask and heated at 300 ºC. At this temperature, Se-Te stock solution (0.25 mL) was quickly injected using a syringe. Immediately, the solution changed from colorless to deeply colored. By monitoring of the growth of QDs with their fluorescence spectra, the formation of the QDs (ca. 780 nm emission) was checked. When the desired QDs were formed, the solution was cooled to 200 ºC. At this temperature, the formation of CdS shell was performed. Addition of the Cd-S stock solution (0.15–0.25 mL) resulted in the formation of the CdSeTe/CdS QDs that emit at 800 nm. Then, the QD solution was cooled to 60 ºC and chloroform (20 mL) was added. The QDs were precipitated by addition of methanol and separated by centrifugation. To remove excess TOPO, HDA, and stearic acid, the QDs were dissolved in chloroform again and precipitated by addition of methanol. This procedure was repeated three times. The resulting QDs were dissolved in tetrahydrofuran (THF, 20 mL) and stored in a dark place.

### 4.3. Preparation of GSH-, MUA- and PEI-coated CdSeTe/CdS QDs

GSH-coated CdSeTe/CdS QDs (GSH-QDs): GSH aqueous solution (100 mg/mL, 0.2 mL) was slowly added to the THF solution (1 μM, 0.5 mL) of CdSeTe/CdS QDs at room temperature and the mixture was heated to 60 ºC. The resulting precipitates of QDs were separated by centrifugation. To the QD precipitates, water (1 mL) was added and then potassium *t*-butoxide (5 mg) was slowly added. The mixture was sonicated for 5 min and filtered through a 0.2 μm membrane filter. Excess GSH and potassium *t*-butoxide were removed by the dialysis using a 10 mM PBS buffer (pH = 7.4).

MUA-coated QDs (MUA-QDs): MUA (10 mg) was added to the THF solution (1 μM, 0.5 mL) of CdSeTe/CdS QDs at room temperature and the mixture was heated to 60 ºC. To this solution potassium *t*-butoxide (5 mg) was slowly added. The resulting precipitates of QDs were separated by centrifugation, and water (1 mL) was added. The solution was sonicated for 5 min and filtered through a 0.2 μm membrane filter. Excess MUA and potassium *t*-butoxide were removed by the dialysis using a 10 mM PBS buffer (pH = 7.4).

PEI-coated QDs (PEI-QDs): PEI (MW. 10,000, 0.5 g) was added to the THF solution (1 μM) of CdSeTe/CdS QDs at room temperature under stirring. Then, the solvent of THF was removed using a rotary evaporator, and water (1 mL) was added. After sonication of the aqueous solution for 5 min, the solution was filtered through a 0.2 μm membrane filter. Excess PEI was removed by the dialysis using a 10 mM PBS buffer (pH = 7.4).

### 4.4. Characterization of QDs

Fluorescence and absorption spectra were recorded at room temperature on a FP-6200 spectrofluorometer (Jasco) and a U-1900 spectrophotometer (Hitachi), respectively. The fluorescence quantum yield (QY) of QDs was measured according to the method described in ref [45]. Indocyanine green (ICG) (with DMSO as the solvent) was chosen as a reference standard (QY = 12%). Measurements of the hydrodynamic diameters were performed using dynamic light scattering (DLS) on Nano-ZS (Malvern) and fluorescence correlation spectroscopy (FCS) on a compact FCS system (C9413-01MOD, Hamamatsu Photonics, Japan). For the determination of the concentration of GSH-QDs, we measured the number of QD particles in 20 μL solution by using FCS [see, eq. (2)], and estimated its concentration using a 20 nM solution of Rhodamine 6G as a reference. For all measurements, pH of the aqueous solution of QDs was set to be 7.4 using PBS buffer.

### 4.5. Cellular uptake and viability test

HeLa and HEK 293 cells were cultured in Dulbecco's modified Eagle's medium (DMEM, Sigma-Aldrich, St. Louis, MO) with 10% fetal bovine serum, 100 U/mL penicillin, and 10 mg/mL streptomycin at 37 ºC (5% CO_2_), and grown in 96-well LabTek chambers (Nalge Nunc International, Rochester, NY). After 48 hrs, the medium was replaced by DMEM (100 μL) containing 10 nM of GSH-QDs, and cells were incubated for 0.5 and 12 hrs at 37 ºC (5% CO_2_). Before measurements of microscopy images, the cells were washed and replaced with Opti-MEM (GIBCO, Paisley, Scotland, UK).

For cell imaging we used an Olympus confocal inverted microscope FluoView1000 equipped with a UPLSAPO 60*×* 1.35 NA oil-immersion objective and differential interference contrast (DIC) system. Hoechst 33342 (Invitrogen, USA) and GSH-QDs were excited at 405 nm with a LD pumped solid laser and detected at 430–455 and 655–755 nm, respectively. ER-Tracker^TM^ Green (Invitorogen, USA) was excited at 473 nm with a LD pumped solid laser and detected at 490–590 nm.

A QMTT reduction assay was performed according to the procedure of a QMTT Cell Viability Assay Kit (BioChain, USA). The solutions of QMTT reagent were added to each well and cells were incubated for 4 hrs at 37 ºC (5% CO_2_). According to the instruction of the Kit, the absorbance at 570 nm (reference at 700 nm) of solubilized QMTT formazan products was measured with an absorption spectrometer (U-1900, Hitachi high-technology corporation, Japan) in a 1-cm path length quartz cell.

### 4.6. In vivo lymph node imaging

A C57BL/6J mouse (9wks, 20 g) was anesthetized with pentobarbital sodium (50 mg/kg). Hairs on the regions of interest were removed by depilatory cream (thioglycolate Calcium). GSH-QDs (1 μM, 100 μL) was carefully injected from the right fore-palm with a syringe, and then the fore-limb was gently massaged by hand to enhance migration of lymphatic fluid for 20 min. The right palm was covered with a piece of aluminium to prevent the intense emission from the region where the QD was injected: the intense emission at the injection site disturbs the signal from the region of interest (i.e. the lymph node). The mouse was put on the fluorescence imaging stage (a prototype *in vivo* imaging apparatus, Shimadzu Co., Japan.) in the supine position. The details of the image acquisition were described in the caption of Figure 9. All experiments were performed in compliance with the National Institutes of Health Guidelines for the Care and Use of Laboratory Animals, and were approved by the Osaka University Animals Care and Use Committee.
